# Ready-to-Use Therapeutic Food (RUTF) Containing Low or No Dairy Compared to Standard RUTF for Children with Severe Acute Malnutrition: A Systematic Review and Meta-Analysis

**DOI:** 10.1093/advances/nmab027

**Published:** 2021-04-10

**Authors:** Isabel Potani, Carolyn Spiegel-Feld, Garyk Brixi, Jaden Bendabenda, Nandi Siegfried, Robert H J Bandsma, André Briend, Allison I Daniel

**Affiliations:** Centre for Global Child Health, Hospital for Sick Children, Toronto, Ontario, Canada; Translational Medicine Program, Hospital for Sick Children, Toronto, Ontario, Canada; Department of Nutritional Sciences, Faculty of Medicine, University of Toronto, Toronto, Ontario, Canada; The Childhood Acute Illness & Nutrition (CHAIN) Network, Blantyre, Malawi; Translational Medicine Program, Hospital for Sick Children, Toronto, Ontario, Canada; Harvard University, Boston, MA, USA; Department of Nutrition for Health and Development, World Health Organization, Geneva, Switzerland; Independent Clinical Epidemiologist, Cape Town, South Africa; Centre for Global Child Health, Hospital for Sick Children, Toronto, Ontario, Canada; Translational Medicine Program, Hospital for Sick Children, Toronto, Ontario, Canada; Department of Nutritional Sciences, Faculty of Medicine, University of Toronto, Toronto, Ontario, Canada; The Childhood Acute Illness & Nutrition (CHAIN) Network, Blantyre, Malawi; Department of Biomedical Sciences, College of Medicine, University of Malawi, Blantyre, Malawi; Center for Child Health Research, University of Tampere School of Medicine, Tampere, Finland; Department of Nutrition, Exercise, and Sports, Faculty of Science, University of Copenhagen, Copenhagen, Denmark; Centre for Global Child Health, Hospital for Sick Children, Toronto, Ontario, Canada; Translational Medicine Program, Hospital for Sick Children, Toronto, Ontario, Canada; Department of Nutritional Sciences, Faculty of Medicine, University of Toronto, Toronto, Ontario, Canada

**Keywords:** meta-analysis, network meta-analysis, meta-regression, severe acute malnutrition, CMAM, protein quality, DIAAS, PDCAAS

## Abstract

Ready-to-use therapeutic food (RUTF) containing less dairy may be a lower-cost treatment option for severe acute malnutrition (SAM). The objective was to understand the effectiveness of RUTF containing alternative sources of protein (nondairy), or <50% of protein from dairy products, compared with standard RUTF in children with SAM. The Cochrane Library, MEDLINE, Embase, CINAHL, and Web of Science were searched using terms relating to RUTF. Studies were eligible if they included children with SAM and evaluated RUTF with <50% of protein from dairy products compared with standard RUTF. Meta-analysis and meta-regression were completed to assess the effectiveness of intervention RUTF on a range of child outcomes. The quality of the evidence across outcomes was assessed using the GRADE (Grading of Recommendations Assessment, Development and Evaluation) approach. A total of 5868 studies were identified, of which 8 articles of 6 studies met the inclusion criteria evaluating 7 different intervention RUTF recipes. Nondairy or lower-dairy RUTF showed less weight gain (standardized mean difference: −0.20; 95% CI: −0.26, −0.15; *P* < 0.001), lower recovery (relative risk ratio: 0.93; 95% CI: 0.87, 1.00; *P* = 0.046), and lower weight-for-age *z* scores (WAZ) near program discharge (mean difference: −0.10; 95% CI: −0.20, 0.0; *P* = 0.047). Mortality, time to recovery, default (consecutive absences from outpatient therapeutic feeding program visits), nonresponse, and other anthropometric measures did not differ between groups. The certainty of evidence was high for weight gain and ranged from very low to moderate for other outcomes. RUTF with lower protein from dairy or dairy-free RUTF may not be as effective as standard RUTF for treatment of children with SAM based on weight gain, recovery, and WAZ evaluated using meta-analysis, although further research is required to explore the potential of alternative formulations. This review was registered at https://www.crd.york.ac.uk/prospero/ as CRD42020160762.

## Introduction

Ready-to-use therapeutic food (RUTF) was developed in 1996 for children with severe acute malnutrition (SAM) based on the composition of F-100, the WHO-recommended diet (1). RUTF is currently used for nutritional rehabilitation of children with SAM in the community and hospital settings per the Community-Based Management of Acute Malnutrition guidelines and has been highly effective in promoting nutritional recovery in children with SAM (2–4). However, these children often remain nutritionally vulnerable, and suffer from other negative long-term effects like impaired linear growth and developmental outcomes (5–8). During SAM recovery, and particularly when children have concurrent infection, which is common in this population, protein requirements are higher compared with periods of normal growth related to catch-up growth (9). Furthermore, SAM is associated with reduced nutrient-absorptive capacity, which can also be exacerbated with infection, and therefore, both protein quality and quantity need to be factored in when addressing protein requirements (10).

Current guidelines state that at least 50% of protein in RUTF should come from milk products as they are of higher protein quality than other ingredients like peanuts (2). It is important to note that milk is a costly ingredient in standard RUTF. Scalability of RUTF may therefore be more achievable with formulations that contain lower milk content, which is a strong rationale for exploring alternative formulations of RUTF. Two systematic reviews have compared reduced-milk RUTF with standard RUTF in terms of weight gain, mortality, and change in anthropometric indices (11, 12). One of the included studies in both of these systematic review meta-analyses evaluated an alternative RUTF consisting of the same amount of milk protein as standard RUTF but made from whey protein as opposed to skim-milk powder, which is likely to have biased the results to show similar outcomes in children consuming alternative RUTF (13). Furthermore, the reviews did not report on other important outcomes such as nonresponse and default rates, defined as consecutive absences from outpatient therapeutic feeding program (OTP) visits, or biochemical and body-composition changes.

It is important to demonstrate if RUTF formulations with <50% of protein from dairy sources are as efficacious, safe, and effective as standard RUTF. The cost-effectiveness of RUTF formulations also requires further exploration to understand if they could potentially allow for wider SAM treatment coverage. This systematic review aimed to answer the following question: “In infants and children aged 6 months or older with uncomplicated SAM, what is the effectiveness of RUTF containing alternative sources of protein (nondairy), or less than 50% of protein coming from dairy products, compared to standard RUTF as specified by WHO (2007) (at least 50% of protein from dairy products)?” A secondary aim was to determine if protein-quality scores of low- or no-dairy RUTF predict weight gain.

## Methods

A protocol for this systematic review was registered on the International Prospective Register of Systematic Reviews (CRD42020160762). The PRISMA (Preferred Reporting Items for Systematic Reviews and Meta-Analyses) guidelines were followed for reporting of this review (14).

### Search strategy

Five electronic databases were searched by a research librarian: the Cochrane Library, MEDLINE, Embase, CINAHL, and Web of Science. Reference lists of included studies were examined to identify additional studies that met the criteria for the review. Trial registries including ClinicalTrials.gov, the ISRCTN registry, and the WHO International Clinical Trials Registry Platform were also searched for studies that had been completed but not yet published, in which case investigators were contacted for results. Conference abstracts and proceedings were identified by searching BIOSIS Previews, which is an index of abstracts and citations from journals, meetings, reports, conferences, and symposia between 1926 and the present. Search terms were broad, with keywords related to RUTF, to identify as many articles as possible on this topic (**Supplemental Table 1**). There were no date, location, or other restrictions for the searches, although no non–English-language articles were identified as being eligible for inclusion in the search.

### Inclusion and exclusion criteria

Studies including infants and children aged 6 mo or older with SAM who had appetite and no medical complications were included. SAM was identified as recommended by the WHO if children met at least 1 of the following criteria (15): *1*) weight-for-height *z* score (WHZ) < −3 SDs, *2*) midupper arm circumference (MUAC) <115 mm, and *3*) bilateral pitting edema.

It is recommended to use RUTF in infants and children with SAM who have been clinically stabilized and initiated on RUTF during inpatient treatment at nutritional rehabilitation units (rehabilitation phase) or during outpatient care (in an OTP). Only studies using these criteria were included, while studies examining only infants and children with moderate acute malnutrition were excluded from this review.

The intervention of interest was RUTF containing <50% of protein from dairy products compared with standard RUTF containing at least 50% of protein from milk and other dairy products. Randomized controlled trials (RCTs) and cluster-RCTs comparing these 2 types of RUTF were eligible and included in the quantitative analysis of this systematic review. However, other nonrandomized trials that compared RUTF containing <50% of protein from dairy products versus RUTF containing at least 50% of protein from milk and other dairy products were included in the review but presented separately from RCTs due to potential biases.

### Relevant child outcomes

There were 5 main outcome categories of interest for this systematic review to assess efficacy, safety, and effectiveness of RUTF with <50% of protein from dairy products (**Table 1**). Additional secondary outcomes considered were body composition, cardiometabolic indices such as glucose tolerance, biochemical assessment, morbidity and other adverse effects, and neurodevelopment.

**TABLE 1:** Main child outcomes of interest for this systematic review of ready-to-use therapeutic food containing low or no dairy for children with severe acute malnutrition^1^

Main outcome categories	Description of main child outcomes
Weight gain	1) Rate of weight gain until recovery (grams per kilogram of body weight per day)
Recovery	1) Recovery (percentage of children cured, defined as midupper arm circumference of at least 125 mm, weight-for-height or -length *z* score of at least −2 SDs, and no edema for at least 2 wk)2) Time to recovery (days until midupper arm circumference of at least 125 mm, weight-for-height or -length *z* score of at least −2 SDs, and no edema for at least 2 wk)3) Sustained recovery (sustained recovery defined as midupper arm circumference of at least 125 mm, weight-for-height or -length *z* score of at least −2 SDs, and no edema for 1 y)
Mortality	1) Mortality (percentage of children who died)
Other OTP outcomes	1) Default (percentage of children who were absent for 3 consecutive visits if OTP is weekly or absent for 2 consecutive visits if OTP is every 2 wk)2) Nonresponse (percentage of children who have not been cured within 4 mo)3) Relapse (percentage of children who re-enrolled in OTP after being cured)
Anthropometry	1) Weight-for-height or -length *z* score2) Midupper arm circumference3) Weight-for-age *z* score4) Height- or length-for-age *z* score

^1^OTP, outpatient therapeutic feeding program.

### Study selection and data extraction

Titles and abstracts were screened in duplicate by any of 3 reviewers (AID, IP, CS-F) followed by full-text screening using Covidence online software (16). Any discrepancies were resolved by a third reviewer who did not screen the articles initially. Data were extracted in duplicate by the 3 reviewers in a REDCap (Research Electronic Data Capture) database (17). Variables included study design and information, participant characteristics, intervention RUTF properties and ingredients, compliance to RUTF, and main outcomes.

Data were extracted to enable an available case analysis using the total amount of known data for each particular outcome, resembling as close to an intention-to-treat analysis as possible. The degree of missing data was considered in the risk of bias and Grading of Recommendations Assessment, Development and Evaluation (GRADE) assessments as potential causes of heterogeneity. For cluster-RCT data, the design effect was applied to compute an effective sample size based on the number of clusters and mean size of each cluster, outcome data for individual participants, and the intracluster correlation coefficient (18).

For each individual study, relevant experimental intervention groups were pooled into a single group, per the Cochrane Handbook (18). However, weight gain was examined more closely with separate intervention groups for individual trials to confirm whether individual study arms did perform differently.

### Data synthesis and analysis

All statistical analysis was completed using Stata version 16 (StataCorp LP) (19). Random-effects meta-analyses were conducted to pool and compare the effectiveness of low- or no-dairy RUTF formulations versus standard RUTF. Hedges’ *g* standardized mean differences (SMDs) and 95% CIs were calculated for meta-analyses of continuous outcomes (e.g., weight gain). Unstandardized mean differences (MDs) were calculated for anthropometric *z* scores. Risk ratios (RRs) and 95% CIs were calculated for dichotomous outcomes (e.g., mortality). Where interventions from multiarm trial results were considered sufficiently similar, these were analyzed together in the meta-analysis as well as separately by halving the control group sample size. Statistical heterogeneity was assessed based on the chi-square statistic *Q* in the meta-analyses, and inconsistency was evaluated based on *I*² values.

Cumulative meta-analysis was completed for weight gain to indicate if the overall effect size changed as each individual study was added to the meta-analysis, beginning at the earliest study and adding studies by year (20, 21). This approach was done to understand the trends in results over time with the development of new and potentially improved formulations of RUTF with <50% of protein from dairy products. Subgroup and network meta-analyses were also conducted with weight gain as the outcome variable to explore differences between nondairy and low-dairy versions of RUTF. Additional subgroup analyses were planned for the main outcomes if there were enough data, including inpatient versus outpatient RUTF initiation, SAM phenotype, age, HIV status, and region of the study.

Furthermore, meta-regression was conducted to explore the relation between protein quality according to protein digestibility–corrected amino acid score (PDCAAS) or digestible indispensable amino acid score (DIAAS) of alternative RUTF and standard RUTF formulations and the SMD for weight gain. PDCAAS and DIAAS were calculated using methods proposed by the Food and Agriculture Organization in order to enable comparison of protein-quality indicators between the recipes (22). For the PDCAAS calculations, the quantity of each individual amino acid per ingredient was multiplied by the ingredient's digestibility score, summed, and divided by the reference quantity. For DIAAS, the quantity of each individual amino acid per ingredient was multiplied by that specific amino acid's digestibility in that ingredient, summed, and divided by the reference quantity. The quantity of each amino acid present in the ingredient was obtained from the USDA nutrient database (23). True protein digestibility scores for the PDCAAS calculations were obtained from published results (24). True ileal digestibility scores for the DIAAS calculations were obtained from the AmiPig report (25).

Upon computing these scores, random-effects meta-regression was conducted using the *metareg macro* with PDCAAS and DIAAS as predictors of the SMD of weight gain in 2 different versions of the meta-regression. The meta-regression analyses were weighted by the inverse of the variance for the SMD. The meta-regression was fitted with restricted maximum likelihood and *I*^2^ values were computed, representing residual variation between study groups.

### Risk of bias and certainty of evidence

The 3 reviewers independently assessed risk of bias using version 2 of the Cochrane risk-of-bias tool for randomized trials (RoB 2) (26). This includes bias from the randomization process, bias due to deviations from intended interventions, bias due to missing outcome data, bias in measurement of outcomes, and bias in selection of the reported results (26). Additional criteria examined for cluster-RCTs included baseline imbalance, loss of clusters, incorrect analysis, and comparability with RCTs (26).

The GRADE approach was then used to assess the certainty of evidence across studies (27). The outcomes were assessed for within-study risk of bias, directness of evidence, heterogeneity, precision of estimate effects, and risk of publication bias. Each outcome was given a certainty rating of high, moderate, low, or very low based on these criteria (27).

## Results

**FIGURE 1:**
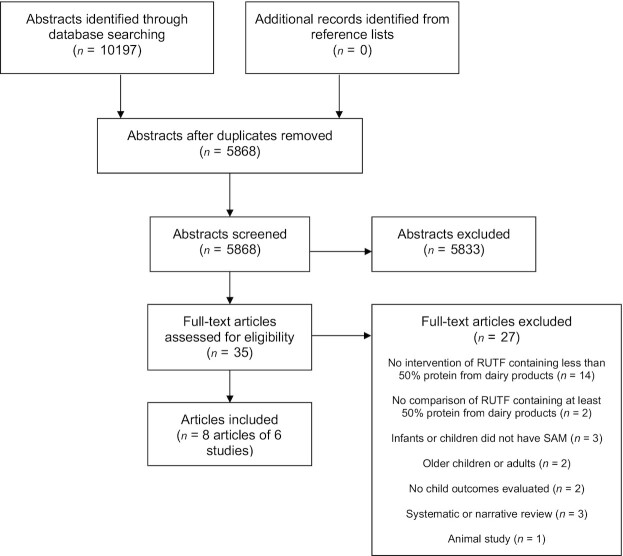
Study flow diagram of articles included and excluded in this systematic review of RUTF containing low or no dairy for children with SAM. RUTF, ready-to-use therapeutic food; SAM, severe acute malnutrition.

The database search on 27 January 2020 yielded 10,197 abstracts, which were narrowed down to 5868 abstracts after duplicates were removed (**Figure 1**). A total of 5833 abstracts were excluded in the first step of screening, which involved examining titles and abstracts. This meant that 35 articles were assessed for eligibility, of which 27 were excluded (**Supplemental Table 2**). Eight articles that presented results for 6 different trials in total were included in this systematic review, with a total sample of 6356 children (28–35).

Forty-four records were identified in the search of ClinicalTrials.gov—18 in the ISRCTN registry and 59 records of 58 trials in the WHO International Clinical Trials Registry Platform—but none of these included trials that were not already captured in the database search. Eighty records were retrieved in the BIOSIS Previews search, but again there were no additional studies that had not been identified in the database search.

Characteristics of the 6 trials included in the systematic review are summarized in **Table 2**. There were several different recipes of RUTF assessed within these trials. The study done by Bahwere et al. (30) in Malawi included 2 different alternative versions of RUTF, both fortified with crystalline amino acids, but one was a milk-free version of RUTF and the other a reduced-milk version. Other versions of RUTF without milk powder were examined by Bahwere et al. (31) in the Democratic Republic of the Congo, Hossain et al. (32) in Bangladesh, Irena et al. (33) in Zambia, and Sigh et al. (35) in Cambodia. In the Sigh et al. (35) study, fish-based RUTF was examined as another form of animal protein. There were 2 versions of RUTF that contained skim-milk powder but in lower quantities than standard RUTF, including the other alternative version of RUTF in the Bahwere et al. (30) study in Malawi, which had 9% skim-milk powder and the Oakley et al. (34) study also conducted in Malawi, which looked at RUTF with 10% skim-milk powder. All were RCTs apart from the Irena et al. (33) study, which was a cluster-RCT with clustering at the health-center level, including a total of 24 health centers that were divided between the 2 trial arms. The intraclass correlation coefficient was 0.015 for the intention-to-treat analysis.

**TABLE 2:** Characteristics of studies evaluating RUTF containing low or no dairy for children with SAM included in the systematic review^1^

			Baseline sample size, *n*		Age range, mo	RUTF <50%^2^	
Study (reference)	Country setting	Study design		SAM types		Description	Protein quality
Oakley 2010 (34)	Malawi (low-income)	RCT	Intervention: 929Comparison: 945	Severe wasting and edematous malnutrition	6–59	Soya RUTF containing 10% skim-milk powder	PDCAAS: 87DIAAS: 54
Irena 2015 (33)	Zambia (low-income)	Cluster-RCT (clustering at the level of the health center, 24 health centers)	Intervention: 824 (effective sample size 376)Comparison: 1103 (effective sample size 504)	Severe wasting and edematous malnutrition	6–59	Soya, maize, and sorghum RUTF without milk/dairy powder	PDCAAS: 84DIAAS: 81
Bahwere 2016 (31)	Democratic Republic of the Congo (low-income)	RCT	Intervention: 439Comparison: 436	Severe wasting and edematous malnutrition	6–59	Soya, maize, and sorghum RUTF without milk/dairy powder	PDCAAS: 91DIAAS: 79
Bahwere 2017 (30)/Sato 2018 (29)/Akomo 2019 (28)	Malawi (low-income)	RCT	Intervention 1: 433Intervention 2: 420Comparison: 446	Severe wasting and edematous malnutrition	6–59	Intervention 1: soya, maize, and sorghum RUTF enriched with crystalline amino acids without milk/dairy powder (FSMS)Intervention 2: soya, maize, and sorghum RUTF enriched with crystalline amino acids containing 9% skim-milk powder (MSMS)	Intervention 1: PDCAAS: 112Intervention 1 DIAAS: 88Intervention 2: PDCAAS: 112Intervention 2 DIAAS: 95
Sigh 2018 (35)	Cambodia (middle-income)	RCT	Intervention: 60Comparison: 61	Severe wasting and edematous malnutrition	6–59	Fish-based wafer RUTF without milk/dairy powder	PDCAAS: 94^3^DIAAS: 85^3^
Hossain 2019 (32)	Bangladesh (middle-income)	RCT	Intervention: 130Comparison: 130	Severe wasting only	6–59	Soya RUTF without milk/dairy powder	Missing information

1 DIAAS, digestible indispensable amino acid score; PDCAAS, protein digestibility–corrected amino acid score; RCT, randomized controlled trial; RUTF, ready-to-use therapeutic food; SAM, severe acute malnutrition.

2 RUTF <50% represents RUTF with <50% of protein coming from dairy products.

3 Animal protein digestibility was used as the best estimate of protein quality for the local fish ingredient.

### Risk of bias of included studies

The overall risk of bias for 3 studies was low, but there were some concerns for the Hossain et al. (32) study and there was high risk of bias for the Sigh et al. (35) and Irena et al. (33) studies.

The Sigh et al. (35) study had high risk of performance and selection bias because children could be re-allocated to different study arms after randomization. Four participants per arm transferred to the other respective arm of the trial in the case that they failed an appetite test for either RUTF. Participants and study personnel were unblinded, which could have increased the risk of performance bias (35). The Irena et al. (33) study also had high risk of bias due to similar issues, as study personnel and participants were unblinded and indicated greater preference for standard RUTF, leading to 43 children switching from the intervention arm to the comparison arm. Both per-protocol and intention-to-treat analyses were completed.

The loss to follow-up for the Sigh et al. (35) study was high, at 38% in the nondairy RUTF group and 34% in the standard RUTF group, meaning that there were some concerns of attrition. There was also high risk of attrition bias due to missing data for the Hossain et al. (32) study, which had a 49% loss to follow-up rate for the dairy-free RUTF group and a 45% loss to follow-up for the standard RUTF group.

There were some concerns of bias due to outcome measurement for all studies because study personnel assessing outcomes were unblinded, apart from the Oakley et al. (34) and Hossain et al. (32) studies, which had low risk of bias because of blinding of study personnel. The bias due to outcome measurement did not differ for each outcome in the various studies.

For the Irena et al. (33) study, which was the only cluster-RCT, there was a high risk of recruitment bias because it appeared that a greater number of children attended health centers supplying standard RUTF, meaning more were recruited to this arm of the trial. There was low risk for baseline imbalance, loss of clusters, incorrect analysis, and comparability with RCTs (33).

### Main child outcomes

The certainty of the evidence for weight gain was rated high according to the GRADE approach; moderate for recovery, nonresponse, weight-for-age *z* scores (WAZ), and height-for-age *z* scores (HAZ); low for mortality, default, WHZ, and MUAC; and very low for time to recovery (**Table 3**).

**TABLE 3:** GRADE evidence profile for outcomes of this systematic review of RUTF containing low or no dairy for children with severe acute malnutrition^1^

	Quality assessment						Number of participants		Effect		
No. of studies	Design	Risk of bias	Inconsistency	Indirectness	Imprecision	Other considerations	RUTF <50%^2^	Standard RUTF	Relative (95% CI)	Absolute	Quality
Weight gain (better indicated by higher values)											
6	RCTs and cluster-RCTs	No serious risk of bias	No serious inconsistency	No serious indirectness	No serious imprecision	None	2366	2185	—	SMD 0.20 lower (0.26 to 0.15 lower)	⊕⊕⊕⊕ High
Recovery											
4	RCTs and cluster-RCTs	No serious risk of bias	No serious inconsistency	Serious^3^	No serious imprecision	None	1941/2597 (74.7%)	1811/2328 (77.8%)	RR 0.93 (0.87 to 1.00)	54 fewer per 1000 (from 101 fewer to 0 fewer)	⊕⊕⊕Ο Moderate
Time to recovery (better indicated by lower values)											
4	RCTs and cluster-RCTs	Serious^4^	Serious^5^	Serious^3^	Serious^6^	None	1292	1129	—	SMD 0.20 higher (0.01 lower to 0.41 higher)	⊕ΟΟΟ Very low
Mortality											
5	RCTs and cluster-RCTs	Serious^4^	No serious inconsistency	No serious indirectness	Serious^6^	None	111/2635 (4.2%)	108/2369 (4.6%)	RR 1.11 (0.85 to 1.44)	5 more per 1000 (from 6 fewer to 20 more)	⊕⊕ΟΟ Low
Default											
3	RCTs and cluster-RCTs	Serious^4^	No serious inconsistency	No serious indirectness	Serious^6^	None	307/1668 (18.4%)	240/1384 (17.3%)	RR 1.16 (0.99 to 1.35)	28 more per 1000 (from 2 fewer to 61 more)	⊕⊕ΟΟ Low
Nonresponse											
4	RCTs and cluster-RCTs	No serious risk of bias	No serious inconsistency	No serious indirectness	Serious^6^	None	151/2633 (5.7%)	108/2332 (4.6%)	RR 1.36 (0.95 to 1.94)	17 more per 1000 (from 24 fewer to 44 more)	⊕⊕⊕Ο Moderate
Weight-for-height *z* scores (better indicated by higher values)											
4	RCTs	Serious^4^	No serious inconsistency	No serious indirectness	Serious^6^	None	1099	1117	—	MD 0.01 higher (0.12 lower to 0.14 higher)	⊕⊕ΟΟ Low
Midupper arm circumference (better indicated by higher values)											
4	RCTs	No serious risk of bias	Serious^5^	Serious^7^	No serious imprecision	None	1099	1117	—	MD 0.06 lower (0.25 lower to 0.13 higher)	⊕⊕ΟΟ Low
Weight-for-age *z* scores (better indicated by higher values)											
3	RCTs	No serious risk of bias	No serious inconsistency	No serious indirectness	Serious^6^	None	1063	1079	—	MD 0.10 lower (0.20 lower to 0 higher)	⊕⊕⊕Ο Moderate
Height-for-age *z* scores (better indicated by higher values)											
4	RCTs	No serious risk of bias	No serious inconsistency	No serious indirectness	Serious^6^	None	1099	1117	—	MD 0.02 lower (0.10 lower to 0.05 higher)	⊕⊕⊕Ο Moderate

1 GRADE, Grading of Recommendations Assessment, Development and Evaluation; MD, mean difference; RCT, randomized controlled trial; RR, risk ratio; RUTF, ready-to-use therapeutic food; SMD, standardized mean difference.

2 Definition of recovery varies between studies.

3 The amount of data from studies with a high risk of bias may affect the interpretation of results.

4 Substantial unexplained statistical heterogeneity.

5 Wide CI around the estimate of the effect.

6 Differences in outcome measurement (change over time and absolute values).

7 RUTF <50% represents RUTF with <50% of protein coming from dairy products.

#### Weight gain

**FIGURE 2:**
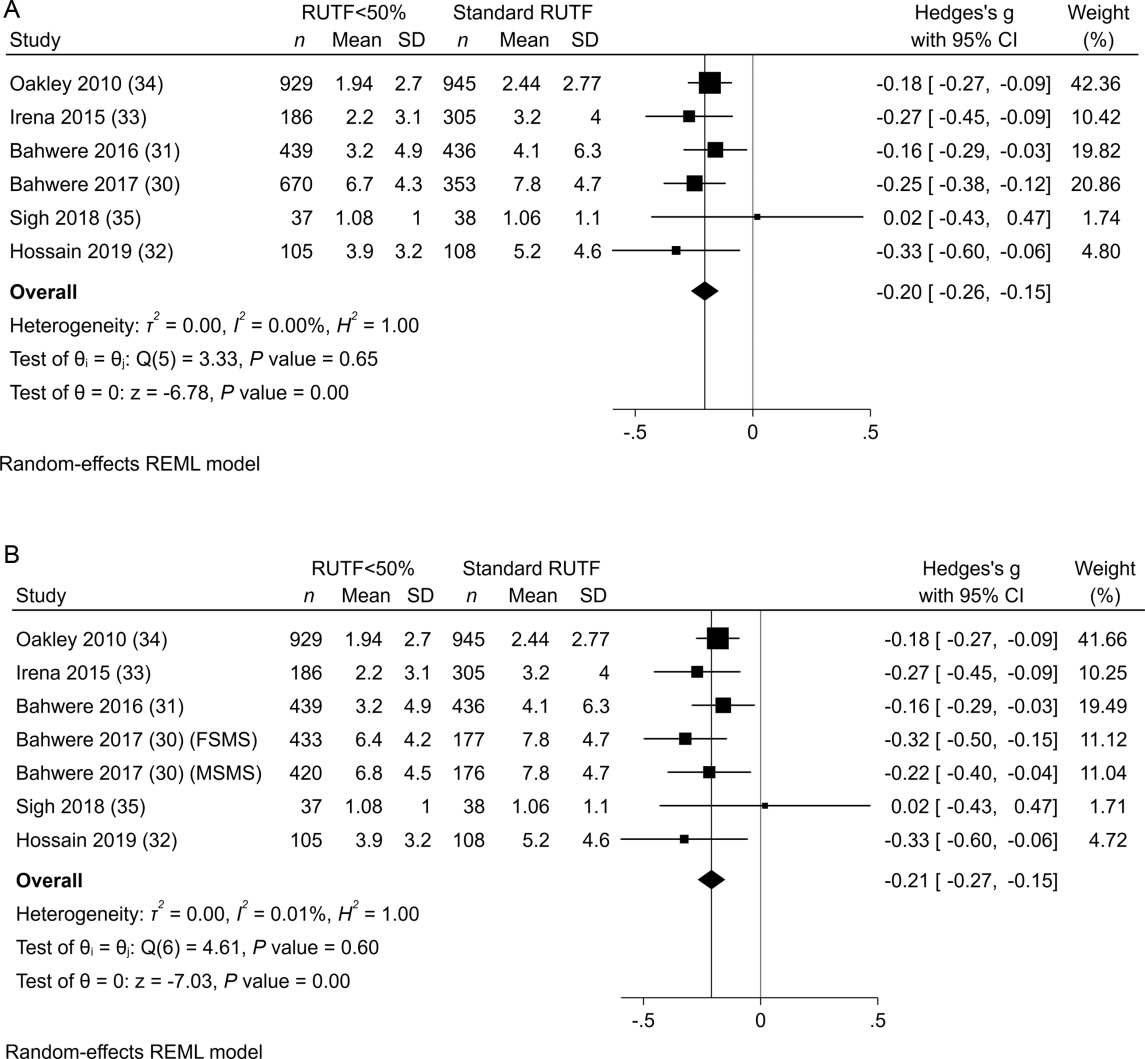
Meta-analysis of the rate of weight gain in grams per kilogram of body weight per day with pooled (A) or separate (B) intervention arms in studies evaluating RUTF containing low or no dairy for children with severe acute malnutrition. RUTF <50% represents RUTF with <50% of protein coming from dairy products. FSMS, milk-free soya, maize, and sorghum; MSMS, milk, soya, maize, and sorghum; REML, restricted maximum likelihood; RUTF, ready-to-use therapeutic food.

The rate of weight gain in grams per kilogram of body weight per day was assessed in all included studies (30–35). For all individual studies, apart from the Sigh et al. (35) study, weight gain was significantly lower in children who consumed RUTF with <50% of protein coming from dairy products compared with standard RUTF based on Hedges’ *g* effect sizes. The meta-analysis results showed that the overall rate of weight gain was significantly lower in children who were given RUTF with <50% of protein from dairy products compared with standard RUTF when intervention groups for the multiarm trial were combined (SMD: −0.20; 95% CI: −0.26, −0.15; *P* < 0.001; *I*^2^ = 0.00%) (**Figure 2**A) or considered separately (SMD: −0.21; 95% CI: −0.27, −0.15; *P* < 0.001; *I*^2^ = 0.01%) (Figure 2B). When excluding the Sigh et al. (35) study, the only trial with fish as an alternative source of protein to dairy, the meta-analysis estimates were similar (SMD: −0.20; 95% CI: −0.27, −0.15; *P* < 0.001; *I*^2^ = 0.0%).

**FIGURE 3:**
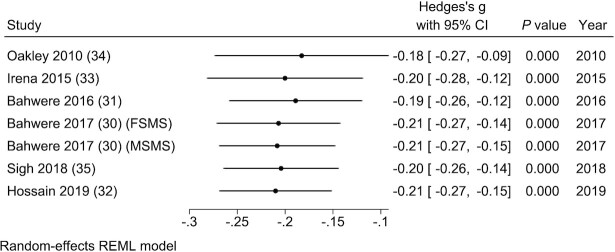
Cumulative meta-analysis of the rate of weight gain in grams per kilogram of body weight per day in studies evaluating ready-to-use therapeutic food containing low or no dairy for children with severe acute malnutrition. FSMS, milk-free soya, maize, and sorghum; MSMS, milk, soya, maize, and sorghum; REML, restricted maximum likelihood.

The cumulative meta-analysis showed that the effect size remained similar over time (**Figure 3**). Furthermore, the subgroup meta-analysis showed that children consuming nondairy and low-dairy versions of RUTF had lower weight gain with similar effect sizes (SMD: −0.23; 95% CI: −0.32, −0.14; *I*^2^ = 7.79%; and SMD: −0.19; 95% CI: −0.27, −0.11; *I*^2^ = 0.01%, respectively) (**Supplemental Figure 1**). These data were also supported by the network meta-analysis (**Supplemental Figure 2**).

#### Recovery

Four studies included in the review reported recovery rates (30, 31, 33, 34). In the Oakley et al. (34) study, recovery was defined as WHZ above −2 and no edema. The Irena et al. (33) study defined recovery as weight gain of at least 18%, MUAC >110 mm, and absence of edema. Neither of the Bahwere et al. studies (30, 31) gave definitions of recovery, but for the Bahwere et al. (31) study published in 2016, investigators defined the discharge criteria as an MUAC of at least 125 mm and no edema for 15 consecutive days.

**FIGURE 4:**
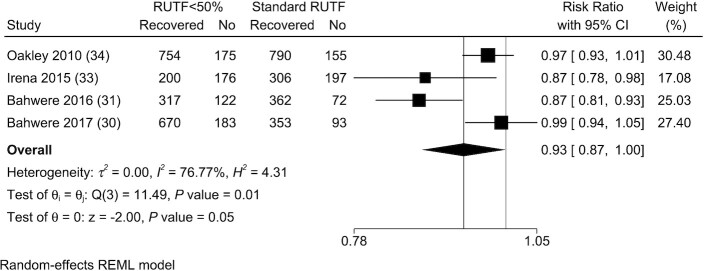
Meta-analysis of recovery in studies evaluating RUTF containing low or no dairy for children with severe acute malnutrition. RUTF <50% represents RUTF with <50% of protein coming from dairy products. REML, restricted maximum likelihood; RUTF, ready-to-use therapeutic food.

The evidence was mixed for individual studies, with 2 studies, including the Irena et al. (33) and Bahwere et al. (31) studies, showing lower recovery in children consuming RUTF with <50% of protein from dairy products. The other 2 studies, by Oakley et al. (34) and Bahwere et al. (30), showed similar recovery rates based on RRs. Overall, the evidence showed that children consuming RUTF with <50% of protein from dairy products resulted in fewer children recovering compared with children consuming standard RUTF (RR: 0.93; 95% CI: 0.87, 1.00; *P* = 0.046; *I*^2^ = 76.77%) (**Figure 4**).

Four studies also examined the duration of time in days until recovery between children (30–33). The Irena et al. (33) study showed that the dairy-free RUTF was associated with a longer time to recovery compared with standard RUTF, but the other 3 studies did not show differences between arms. The meta-analysis results indicated that the length of stay in treatment could be longer in children consuming RUTF with <50% of protein from dairy products, although the difference was not significant (SMD: 0.20; 95% CI: −0.01, 0.41; *P* = 0.06; *I*^2^ = 83.27%) (**Supplemental Figure 3**).

#### Mortality

**FIGURE 5:**
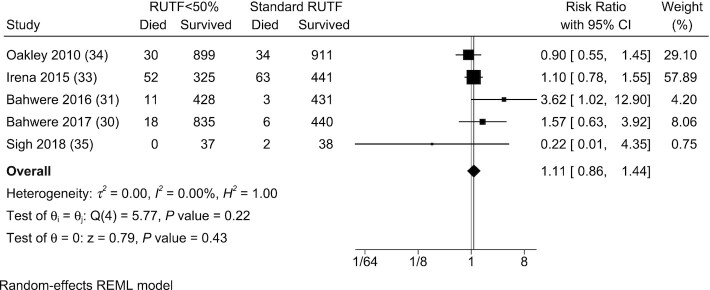
Meta-analysis of mortality in studies evaluating RUTF containing low or no dairy for children with severe acute malnutrition. RUTF <50% represents RUTF with <50% of protein coming from dairy products. REML, restricted maximum likelihood; RUTF, ready-to-use therapeutic food.

Five of 6 included studies documented mortality within their trials (30, 31, 33–35). One study showed a statistically significant difference in mortality, with higher mortality in children consuming RUTF with <50% of protein from dairy products versus standard RUTF in the meta-analysis (31). The remaining 4 studies did not show differences in mortality between groups. The RR of mortality did not differ between groups based on the meta-analysis (RR: 1.11; 95% CI: 0.86, 1.44; *P* = 0.4; *I*^2^ = 0.0%) (**Figure 5**).

#### Other OTP outcomes

Three included studies examined the number of children who defaulted and 4 assessed nonresponse, while none documented relapse (30, 31, 33, 34). The Oakley et al. (34) study documented referral to inpatient treatment, which was 4% in children in the low-milk RUTF group compared with 2% in children in the standard RUTF group (*P* = 0.01) (34).

**FIGURE 6:**
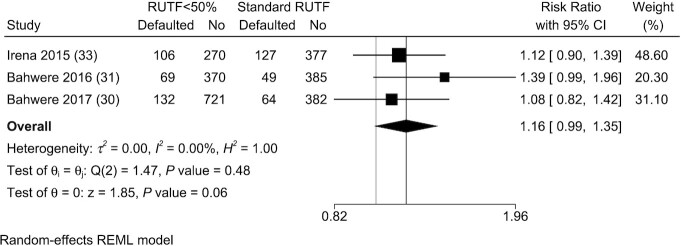
Meta-analysis of default in studies evaluating RUTF containing low or no dairy for children with severe acute malnutrition. RUTF <50% represents RUTF with <50% of protein coming from dairy products. REML, restricted maximum likelihood; RUTF, ready-to-use therapeutic food.

There were no significant differences between groups in terms of the proportion of children who defaulted in individual trials. The pooled data in the meta-analysis showed that children between groups were similar in terms of default rates based on statistical analysis (RR: 1.16; 95% CI: 0.99, 1.35; *P* = 0.06; *I*^2^ = 0.0%) (**Figure 6**).

**FIGURE 7:**
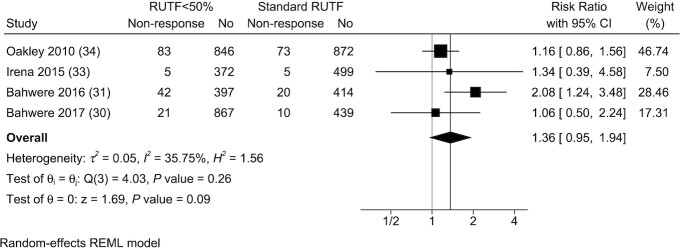
Meta-analysis of nonresponse in studies evaluating RUTF containing low or no dairy for children with severe acute malnutrition. RUTF <50% represents RUTF with <50% of protein coming from dairy products. REML, restricted maximum likelihood; RUTF, ready-to-use therapeutic food.

The Bahwere et al. (31) study showed significantly higher nonresponse in children consuming milk-free RUTF compared with standard RUTF according to RRs, but the other 3 did not show significant differences. The meta-analysis indicated that RUTF with <50% of protein from dairy products had a higher but nonsignificant risk of nonresponse compared with children consuming standard RUTF (RR: 1.36; 95% CI: 0.95, 1.94; *P* = 0.09; *I*^2^ = 35.8%) (**Figure 7**).

#### Anthropometry

WHZ at follow-up was reported in 4 of the included studies, with no significant differences between groups in individual studies or in the meta-analysis (MD: 0.01; 95% CI: −0.12, 0.14; *P* = 0.9; *I*^2^ = 34.90%) (**Supplemental Figure 4**) (31, 32, 34, 35). Results were similar for MUAC, which was also assessed in 4 different studies, with the Oakley et al. (34) study documenting change in MUAC per day and the others recording MUAC at follow-up (31, 32, 35). MUAC was significantly lower in children who consumed RUTF with <50% of protein from dairy products in the Oakley et al. (34) study, but results were the same between groups in the meta-analysis (SMD: −0.06; 95% CI: −0.25, −0.13; *P* = 0.5; *I*^2^ = 47.39%) (**Supplemental Figure 5**). WAZ was not significantly different between groups for individual studies. However, in the meta-analysis including 3 studies that assessed these *z* scores at follow-up, children consuming RUTF with <50% of protein from dairy products had lower WAZ at follow-up than those consuming standard RUTF (MD: −0.10; 95% CI: −0.20, 0.0; *P* = 0.047; *I*^2^ = 0.0%) (**Supplemental Figure 6**) (31, 32, 34). Lastly, HAZ at follow-up, which was examined in 4 studies, was similar between groups for individual studies and in the meta-analysis (MD: −0.05; 95% CI: −0.13, 0.04; *P* = 0.3; *I*^2^ = 0.0%) (**Supplemental Figure 7**) (31, 32, 34, 35).

### Secondary outcomes

Two studies examined the effects of low- or no-dairy RUTF on body composition using the deuterium dilution technique and bioimpedance analysis (BIA) (31, 32). In the Bahwere et al. (31) study, children consuming standard RUTF had a significantly higher fat-free mass index than those consuming lower-dairy RUTF (difference: −0.5 kg/m^2^; 95% CI: −0.85, −0.15; *P* = 0.006) based on BIA. The Hossain et al. (32) study showed similar results in terms of body composition between study groups.

Plasma amino acids were assessed in 2 studies across 3 publications, most of which did not differ in children assigned different versions of RUTF (29–31). Plasma cysteine concentrations were lower in children in the Bahwere et al. (31) study consuming the intervention RUTF compared with standard RUTF (24.96 μmol/L; 95% CI: 16.70, 34.08; compared to 35.60 μmol/L; 95% CI: 29.00, 39.04; *P* = 0.004).

Two trials, published in 3 manuscripts, assessed differences in anemia and iron deficiency biomarkers between groups (28, 30, 31). The alternative versions of RUTF with enhanced iron and phytic acid concentrations that were used in the Akomo et al. (28) and Bahwere et al. (30) studies resulted in lower iron deficiency and anemia rates compared with standard RUTF, although improvements in hemoglobin status were seen in intervention and comparison groups. Results were similar in the earlier Bahwere et al. (31) study with a milk-free version of RUTF, which was enhanced with iron and phytic acid, showing that this formulation improved hemoglobin concentration (difference: 0.67 g/dL; 95% CI: 0.42, 0.92; *P* < 0.001).

Several of the included studies reported on morbidity and other adverse outcomes, including fever, diarrhea, cough, abdominal discomfort, and rash (31–33, 35). These outcomes did not differ by arms, except the Bahwere et al. (31) study reported that children consuming the intervention version of RUTF had lower rates of flatulence within children under 2 y of age. None of the included studies reported on cardiometabolic indices or neurodevelopment.

### Protein quality meta-regression

**FIGURE 8:**
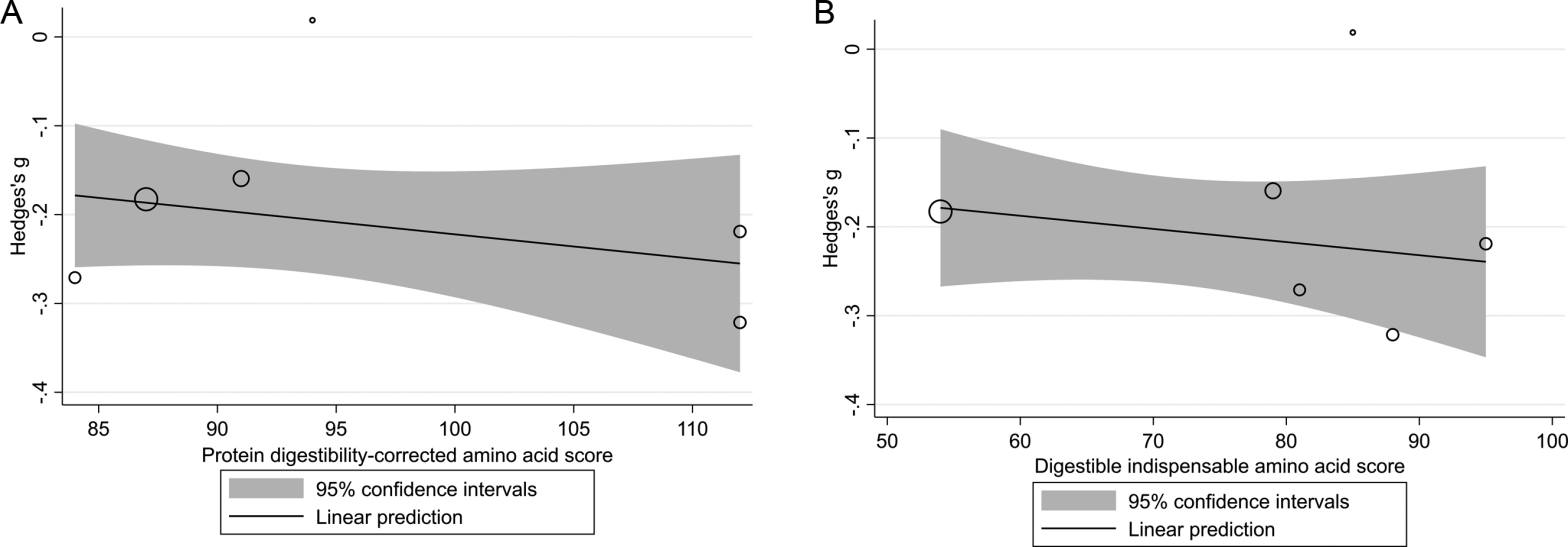
Meta-regression of the relation between protein digestibility–corrected amino acid score (A) and digestible indispensable amino acid score (B) and the standardized mean difference in weight gain, respectively, in studies evaluating ready-to-use therapeutic food containing low or no dairy for children with severe acute malnutrition. The bubble sizes are proportional to the inverse of the variance for the standardized mean difference in weight gain. The solid line represents the linear prediction for the means of weight gain as a function of each of the protein-quality scores.

Meta-regression was conducted with data from the 5 included studies with adequate data for calculation of PDCAAS and DIAAS values (30, 31, 33–35). The Bahwere et al. (30) study included 2 different intervention arms, which were separated for this analysis. The meta-regression analysis showed that neither PDCAAS nor DIAAS were predictive of the SMD for weight gain (β: −0.0027; 95% CI: −0.0084, 0.0031; *P* = 0.4; *I*^2^ = 0.01%; and β: −0.0015; 95% CI: −0.0052, 0.0023; *P* = 0.4; *I*^2^ = 0.0%, respectively) (**Figure 8**).

### Subgroup analyses

There were too few studies looking at similar outcomes to conduct the preplanned subgroup analyses, such as phase of treatment, SAM phenotypes, HIV status, and study region. Firstly, there were no discrete data on children in the rehabilitation phase of inpatient treatment. Only 2 studies looked at results in children with severe wasting versus edematous malnutrition and 3 studies compared children between age categories (30, 31, 33, 34). No studies compared children with HIV with those without HIV. Most of the studies were conducted in Africa and 2 were conducted in Asia, but there were no studies conducted in South America, Western Pacific, or the Eastern Mediterranean regions.

## Discussion

This systematic review summarizes results from 6 different studies examining the effect of 7 versions of RUTF containing alternative sources of protein (nondairy) or <50% of protein coming from dairy products. For several of the main child outcomes, including rate of weight gain and recovery, and WAZ, standard RUTF performed better than lower-dairy RUTF when pooling the data.

The previous systematic review by Das et al. (12) also found standard RUTF to be better than reduced-milk RUTF in terms of weight gain and recovery. As mentioned previously, the meta-analysis by Das et al. (12) included studies with whey protein as alternative versions of RUTF, and therefore showed a smaller effect size compared with our systematic review. The systematic review by Schoonees et al. (11), which compared alternative RUTF formulations although not exclusively reduced-milk formulations, found that there were no differences in terms of weight gain and recovery, but children consuming standard RUTF had lower relapse rates.

Of the anthropometric indices, WAZ was higher in children consuming standard RUTF compared with those given reduced dairy RUTF, although MUAC, WHZ, and HAZ were similar between groups. However, weight gain per day may be a more sensitive indicator of growth within short periods of time compared with anthropometric indices. Again, studies with a longer duration can give greater insight into the results of various RUTF formulations on growth of children with SAM. Beyond this, weight gain and anthropometry are proxy indicators of more important outcomes like child health and development, and further studies evaluating the effects of RUTF formulations on these types of outcomes are needed.

One study included in the systematic review, the Bahwere et al. (31) study, indicated that children had significantly lower fat-free mass based on BIA when consuming RUTF with lower dairy content compared with standard RUTF. These differences were not observed when using the deuterium dilution technique. This distinction also emphasizes the need for more research in understanding the performance of body-composition assessment during SAM, including comparison to community controls.

Achieving better iron status and reducing anemia may result in better long-term outcomes such as improved child development for SAM survivors. Two trials included assessments of anemia and iron biomarkers and favored reduced-dairy RUTF. One of the included studies, published by Akomo et al. (28), attributed this to the inhibitory effect of milk protein on iron absorption. It is also likely that the higher amounts of iron and vitamin C in the intervention RUTF led to improvements in certain biomarkers. However, plant sources of protein that may be used in low- or no-dairy versions of RUTF also contain factors inhibiting iron absorption. The standard RUTF group also had increases in hemoglobin concentration at discharge (28, 30, 31), but it may be insufficient to resolve anemia and iron deficiency (36). There is a need to explore RUTF formulations with varying levels of iron content as well as other means of iron supplementation.

All of the studies included in this review only followed participants up until discharge from OTP or shortly thereafter. Studies with longer follow-up durations of up to 1 y are also needed to fully understand the effects of alternative versions of RUTF on outcomes like anthropometry, body composition, the gut microbiota, as well as child development and overall health. It is possible that the lower rate of weight gain seen in children consuming low-dairy RUTF compared with standard RUTF is not a problem if long-term growth outcomes are achieved, although this is still unknown.

While this systematic review does not include a cost-effectiveness evaluation, reduced-milk RUTF could possibly lower SAM treatment costs, although if these versions of RUTF result in worse outcomes or longer time in treatment then the costs may be similar. This is a strong reason to continue aiming to optimize less-expensive versions of RUTF. Whey protein is a lower-cost dairy alternative to skim-milk powder, although there has been limited research on these versions of RUTF. One study by Bahwere et al. (13) found a whey protein version of RUTF to be similar to standard RUTF in terms of weight gain and recovery, but a more recent study by Kohlmann et al. (37) showed that standard RUTF still performed better in terms of recovery. However, the differences in the Kohlmann et al. (37) study could be attributed to major changes in RUTF composition, including the addition of soy and sorghum, whereas the Bahwere et al. (13) study simply replaced skim-milk powder with whey protein. A potentially lower-cost version of RUTF with whey protein could be a future direction of research.

Additional considerations for research on different formulations of RUTF are the inclusion of subgroups such as children admitted for inpatient care versus children treated only in outpatient care, children with severe wasting versus edematous malnutrition, children in different age categories, and children who are HIV positive or HIV negative. For example, in the Bahwere et al. (31) study, nondairy RUTF was equivalent in recovery for children >24 mo of age but did not achieve equivalency in children <24 mo of age. Furthermore, it is well documented that HIV is associated with poor nutritional outcomes, and therefore RUTF may perform differently in HIV-positive children or those with other acute illnesses or underlying infection ( 38).

### Limitations

An important limitation of this systematic review is the heterogeneity of the low- or no-dairy RUTF formulations that were pooled in the meta-analysis. The rationale for this approach was that the current guidelines include a statement that at least 50% of protein in RUTF should come from milk products (2), although the question requires further evaluation of the different low- or no-dairy RUTF formulations used in the various studies. To account for this, cumulative and network meta-analyses were completed, which did not change the direction or magnitude of the results. Furthermore, there was considerable variability in weight gain even between groups of children consuming standard RUTF across different studies. Meta-regression did not give insight into the relation between protein quality and weight gain, and more research on the influence of factors such as protein quality and source in RUTF is needed. Beyond this, exploration of whether certain nondairy ingredients in RUTF are more effective than others may be useful.

### Conclusions

RUTF with <50% of protein coming from dairy products appears to be comparatively less effective based on weight gain, recovery, and WAZ evaluated using meta-analysis. However, as there was such variability in the formulations, these results should be interpreted with caution. We suggest that future research designs consider longer follow-up periods to assess important outcomes such as child development and should include children in various subgroups. Alternative formulations of RUTF could potentially be optimized with micronutrients and possibly be made less expensive using other ingredients in place of skim milk.

## Supplementary Material

nmab027_Supplemental_File

## Data Availability

Data described in the manuscript, code book, and analytic code will be made available upon request pending application and approval.

## Notes

This systematic review was commissioned and funded by the Evidence and Programme Guidance Unit of the World Health Organization Department of Nutrition for Health and Development and informed a Guideline Development Group meeting. Trainee support was provided by the Canadian Institutes of Health Research (AID).

Author disclosures: The authors report no conflicts of interest.

Supplemental Tables 1 and 2 and Supplemental Figures 1–7 are available from the “Supplementary data” link in the online posting of the article and from the same link in the online table of contents at https://academic.oup.com/advances/.

Abbreviations used: BIA, bioimpedance analysis; DIAAS, digestible indispensable amino acid score; GRADE, Grading of Recommendations Assessment, Development and Evaluation; HAZ, height-for-age *z* score; MD, mean difference; MUAC, midupper arm circumference; OTP, outpatient therapeutic feeding program; PDCAAS, protein digestibility–corrected amino acid score; RCT, randomized controlled trial; RR, risk ratio; RUTF, ready-to-use therapeutic food; SAM, severe acute malnutrition; SMD, standardized mean difference; WAZ, weight-for-age *z* score; WHZ, weight-for-height *z* score.
