# Supplementary material for: Ready-to-Use Therapeutic Food (RUTF) Containing Low or No Dairy Compared to Standard RUTF for Children with Severe Acute Malnutrition: A Systematic Review and Meta-Analysis
Source: Adv Nutr. 2021 Apr 10;12(5):1930–43. doi: 10.1093/advances/nmab027 (PMC8483958; doi:10.1093/advances/nmab027)
Supplement: nmab027_Supplemental_File [file nmab027_supplemental_file.docx]

**Supplemental Table 1**

| **Search: Ready to Use Therapeutic Food** |  |
| --- | --- |
| **Database [Platform]** *Searches run Jan 27, 2020* | **Results** |
| MEDLINE(R) and Epub Ahead of Print, In-Process & Other Non-Indexed Citations and Daily [OVID] *1946 to Jan 24, 2020* | 2399 |
| Embase Classic+Embase [OVID] *1947 to week 4* | 2956 |
| Cochrane Library [Wiley] *Jan 27, 2020* | 1367 |
| CINAHL [EBSCO] *Jan 27, 2020* | 665 |
| Web of Science [Clarivate Analytics] *Jan 27, 2020* | 2811 |
| **TOTAL** | **10,198** |

**Ovid MEDLINE(R) and Epub Ahead of Print, In-Process & Other Non-Indexed Citations and Daily**1946 to January 27, 2020
Search Strategy:

| **#** | **Searches** | **Results** |
| --- | --- | --- |
| 1 | (therapeutic adj3 (food* or diet*)).tw,kf. | 2279 |
| 2 | (("ready-to-use" or "ready to use") adj2 (feed* or food*)).tw,kf. | 283 |
| 3 | (RUTF or RTUF or "plumpy?nut" or "plumpy nut" or chiponde or nourimanba or nutriset or dutasi or "high energy bar").tw,kf. | 143 |
| 4 | or/1-3 | 2399 |

**Embase Classic+Embase**1947 to 2020 Week 04
Search Strategy:

| **#** | **Searches** | **Results** |
| --- | --- | --- |
| 1 | "ready to use therapeutic food"/ | 48 |
| 2 | (therapeutic adj3 (food* or diet*)).tw,kw. | 2781 |
| 3 | (("ready-to-use" or "ready to use") adj2 (feed* or food*)).tw,kw. | 330 |
| 4 | (RUTF or RTUF or "plumpy?nut" or "plumpy nut" or chiponde or nourimanba or nutriset or dutasi or "high energy bar").tw,kw. | 172 |
| 5 | or/1-4 | 2956 |

**Cochrane Library (Wiley)**

Date Run: 27/01/2020 15:27:23

ID Search Hits

#1 ((*therapeutic near/3 (food* or diet*))):ti,ab,kw (Word variations have been searched) 1275

#2 ("ready to use" near/2 (feed* or food*)):ti,ab,kw (Word variations have been searched) 182

#3 ("ready-to-use" near/2 (feed* or food*)):ti,ab,kw (Word variations have been searched) 0

#4 (RUTF or RTUF or "plumpy?nut" or "plumpy nut" or chiponde or nourimanba or nutriset or dutasi or "high energy bar"):ti,ab,kw (Word variations have been searched) 116

#5 #1 OR #2 OR #3 OR #4 1367

**CINAHL [EBSCO]**

Jan 27, 2027

| Monday, January 27, 2020 2:44:13 PM |
| --- |

| **#** | **Query** | **Limiters/Expanders** | **Last Run Via** | **Results** |
| --- | --- | --- | --- | --- |
| S9 | S1 OR S2 OR S3 OR S4 OR S5 OR S6 OR S7 OR S8 | Search modes - Boolean/Phrase | Interface - EBSCOhost Research Databases Search Screen - Advanced Search Database - CINAHL | 665 |
| S8 | TI ( chiponde or nourimanba or nutriset or dutasi or "high energy bar" ) OR AB ( chiponde or nourimanba or nutriset or dutasi or "high energy bar" ) | Search modes - Boolean/Phrase | Interface - EBSCOhost Research Databases Search Screen - Advanced Search Database - CINAHL | 7 |
| S7 | TI "plumpy nut" OR AB "plumpy nut" | Search modes - Boolean/Phrase | Interface - EBSCOhost Research Databases Search Screen - Advanced Search Database - CINAHL | 1 |
| S6 | TX Plumpy'Nut | Search modes - Boolean/Phrase | Interface - EBSCOhost Research Databases Search Screen - Advanced Search Database - CINAHL | 11 |
| S5 | TI plump?nut OR AB plump?nut | Search modes - Boolean/Phrase | Interface - EBSCOhost Research Databases Search Screen - Advanced Search Database - CINAHL | 0 |
| S4 | TI ( RUTF or RTUF ) OR AB ( RUTF or RTUF ) | Search modes - Boolean/Phrase | Interface - EBSCOhost Research Databases Search Screen - Advanced Search Database - CINAHL | 64 |
| S3 | TI ( ("ready-to-use" N2 (feed* or food*)) ) OR AB ( ("ready-to-use" N2 (feed* or food*)) ) | Search modes - Boolean/Phrase | Interface - EBSCOhost Research Databases Search Screen - Advanced Search Database - CINAHL | 146 |
| S2 | TI ( ("ready to use" N2 (feed* or food*)) ) OR AB ( ("ready to use" N2 (feed* or food*)) ) | Search modes - Boolean/Phrase | Interface - EBSCOhost Research Databases Search Screen - Advanced Search Database - CINAHL | 146 |
| S1 | TI ( (therapeutic N3 (food* or diet*)) ) OR AB ( (therapeutic N3 (food* or diet*)) ) | Search modes - Boolean/Phrase | Interface - EBSCOhost Research Databases Search Screen - Advanced Search Database - CINAHL | 603 |

**Web of Science [Clarivate Analytics]**

Jan 27, 2020

| # 9 | [**2,811**](http://apps.webofknowledge.com.myaccess.library.utoronto.ca/summary.do?product=WOS&doc=1&qid=15&SID=6C99TvL1CZeaNBy6SAz&search_mode=CombineSearches&update_back2search_link_param=yes) | #8 OR #7 OR #6 OR #5 OR #4 OR #3 OR #2 OR #1  *Indexes=SCI-EXPANDED, SSCI, A&HCI, CPCI-S, CPCI-SSH, BKCI-S, BKCI-SSH, ESCI Timespan=All years* | [Edit](http://apps.webofknowledge.com.myaccess.library.utoronto.ca/WOS_AdvancedSearch_input.do?product=WOS&SID=6C99TvL1CZeaNBy6SAz&search_mode=AdvancedSearch&replaceSetId=9&editState=init) |  |  |
| --- | --- | --- | --- | --- | --- |
|  | | | | | |
| # 8 | [**14**](http://apps.webofknowledge.com.myaccess.library.utoronto.ca/summary.do?product=WOS&doc=1&qid=11&SID=6C99TvL1CZeaNBy6SAz&search_mode=AdvancedSearch&update_back2search_link_param=yes) | TS=(chiponde or nourimanba or nutriset or dutasi or "high energy bar")  *Indexes=SCI-EXPANDED, SSCI, A&HCI, CPCI-S, CPCI-SSH, BKCI-S, BKCI-SSH, ESCI Timespan=All years* | [Edit](http://apps.webofknowledge.com.myaccess.library.utoronto.ca/WOS_AdvancedSearch_input.do?product=WOS&SID=6C99TvL1CZeaNBy6SAz&search_mode=AdvancedSearch&replaceSetId=8&editState=init) |  |  |
|  | | | | | |
| # 7 | [**21**](http://apps.webofknowledge.com.myaccess.library.utoronto.ca/summary.do?product=WOS&doc=1&qid=10&SID=6C99TvL1CZeaNBy6SAz&search_mode=AdvancedSearch&update_back2search_link_param=yes) | TS=("plumpy nut")  *Indexes=SCI-EXPANDED, SSCI, A&HCI, CPCI-S, CPCI-SSH, BKCI-S, BKCI-SSH, ESCI Timespan=All years* | [Edit](http://apps.webofknowledge.com.myaccess.library.utoronto.ca/WOS_AdvancedSearch_input.do?product=WOS&SID=6C99TvL1CZeaNBy6SAz&search_mode=AdvancedSearch&replaceSetId=7&editState=init) |  |  |
|  | | | | | |
| # 6 | [**21**](http://apps.webofknowledge.com.myaccess.library.utoronto.ca/summary.do?product=WOS&doc=1&qid=9&SID=6C99TvL1CZeaNBy6SAz&search_mode=AdvancedSearch&update_back2search_link_param=yes) | TS=("plumpy'nut")  *Indexes=SCI-EXPANDED, SSCI, A&HCI, CPCI-S, CPCI-SSH, BKCI-S, BKCI-SSH, ESCI Timespan=All years* | [Edit](http://apps.webofknowledge.com.myaccess.library.utoronto.ca/WOS_AdvancedSearch_input.do?product=WOS&SID=6C99TvL1CZeaNBy6SAz&search_mode=AdvancedSearch&replaceSetId=6&editState=init) |  |  |
|  | | | | | |
| # 5 | [**2**](http://apps.webofknowledge.com.myaccess.library.utoronto.ca/summary.do?product=WOS&doc=1&qid=7&SID=6C99TvL1CZeaNBy6SAz&search_mode=AdvancedSearch&update_back2search_link_param=yes) | TS=(plumpy*nut)  *Indexes=SCI-EXPANDED, SSCI, A&HCI, CPCI-S, CPCI-SSH, BKCI-S, BKCI-SSH, ESCI Timespan=All years* | [Edit](http://apps.webofknowledge.com.myaccess.library.utoronto.ca/WOS_AdvancedSearch_input.do?product=WOS&SID=6C99TvL1CZeaNBy6SAz&search_mode=AdvancedSearch&replaceSetId=5&editState=init) |  |  |
|  | | | | | |
| # 4 | [**132**](http://apps.webofknowledge.com.myaccess.library.utoronto.ca/summary.do?product=WOS&doc=1&qid=4&SID=6C99TvL1CZeaNBy6SAz&search_mode=AdvancedSearch&update_back2search_link_param=yes) | TS=(RUTF OR RTUF)  *Indexes=SCI-EXPANDED, SSCI, A&HCI, CPCI-S, CPCI-SSH, BKCI-S, BKCI-SSH, ESCI Timespan=All years* | [Edit](http://apps.webofknowledge.com.myaccess.library.utoronto.ca/WOS_AdvancedSearch_input.do?product=WOS&SID=6C99TvL1CZeaNBy6SAz&search_mode=AdvancedSearch&replaceSetId=4&editState=init) |  |  |
|  | | | | | |
| # 3 | [**340**](http://apps.webofknowledge.com.myaccess.library.utoronto.ca/summary.do?product=WOS&doc=1&qid=3&SID=6C99TvL1CZeaNBy6SAz&search_mode=AdvancedSearch&update_back2search_link_param=yes) | TS=("ready-to-use" NEAR/2 (feed* or food*))  *Indexes=SCI-EXPANDED, SSCI, A&HCI, CPCI-S, CPCI-SSH, BKCI-S, BKCI-SSH, ESCI Timespan=All years* | [Edit](http://apps.webofknowledge.com.myaccess.library.utoronto.ca/WOS_AdvancedSearch_input.do?product=WOS&SID=6C99TvL1CZeaNBy6SAz&search_mode=AdvancedSearch&replaceSetId=3&editState=init) |  |  |
|  | | | | | |
| # 2 | [**340**](http://apps.webofknowledge.com.myaccess.library.utoronto.ca/summary.do?product=WOS&doc=1&qid=2&SID=6C99TvL1CZeaNBy6SAz&search_mode=AdvancedSearch&update_back2search_link_param=yes) | TS=("ready to use" NEAR/2 (feed* or food*))  *Indexes=SCI-EXPANDED, SSCI, A&HCI, CPCI-S, CPCI-SSH, BKCI-S, BKCI-SSH, ESCI Timespan=All years* | [Edit](http://apps.webofknowledge.com.myaccess.library.utoronto.ca/WOS_AdvancedSearch_input.do?product=WOS&SID=6C99TvL1CZeaNBy6SAz&search_mode=AdvancedSearch&replaceSetId=2&editState=init) |  |  |
|  | | | | | |
| # 1 | [**2,668**](http://apps.webofknowledge.com.myaccess.library.utoronto.ca/summary.do?product=WOS&doc=1&qid=14&SID=6C99TvL1CZeaNBy6SAz&search_mode=AdvancedSearch&update_back2search_link_param=yes) | TS=(therapeutic NEAR/3 (food* or diet*))  *Indexes=SCI-EXPANDED, SSCI, A&HCI, CPCI-S, CPCI-SSH, BKCI-S, BKCI-SSH, ESCI Timespan=All years* | [Edit](http://apps.webofknowledge.com.myaccess.library.utoronto.ca/WOS_AdvancedSearch_input.do?product=WOS&SID=6C99TvL1CZeaNBy6SAz&search_mode=AdvancedSearch&replaceSetId=1&editState=init) |  |  |

**Supplemental Table 2.** Excluded studies.

| **Study** | **Reason for exclusion** |
| --- | --- |
| Akram 2016 (44) | No intervention of RUTF containing less than 50% of protein from dairy products (high-density diet including milk powder) |
| Bahwere 2014 (18) | No intervention of RUTF containing less than 50% of protein from dairy products (milk whey protein RUTF) |
| Bhandari 2016 (45) | No intervention of RUTF containing less than 50% of protein from dairy products (RUTF with similar milk composition) |
| Borg 2019 (46) | Systematic or narrative review (summary of locally produced RUTF) and no child outcomes evaluated |
| Brown 2015 (47) | No intervention of RUTF containing less than 50% of protein from dairy products (bigh-density diet including milk powder) |
| Chauhan 2019 (48) | No comparison to RUTF containing at least 50% of protein from milk and other dairy products (standard RUTF) |
| Choudhury 2018 (49) | No child outcomes evaluated (acceptability study only) |
| Dibari 2013 (50) | Adults or older children (age over 18 years) and no child outcomes evaluated (acceptability study) |
| Dube 2009 (51) | No comparison to RUTF containing at least 50% of protein from milk and other dairy products (standard RUTF) |
| Fatima 2017 (52) | Infants/children without SAM (focused on mildly underweight children) |
| Hassan 2016 (53) | Animal study (not human research) |
| Hsieh 2015 (54) | No intervention of RUTF containing less than 50% of protein from dairy products (RUTF with milk whey and dry skimmed milk) |
| Huynh Phuong 2014 (55) | No intervention of RUTF containing less than 50% of protein from dairy products (RUTF with similar milk composition) and no child outcomes evaluated |
| Jadhav 2019 (56) | No intervention of RUTF containing less than 50% of protein from dairy products (RUTF with similar milk composition) |
| Jones 2015 (57) | No intervention of RUTF containing less than 50% of protein from dairy products (RUTF with similar milk composition) |
| Kohlmann 2019 (42) | No intervention of RUTF containing less than 50% of protein from dairy products (milk whey protein RUTF) |
| Manary 2013 (58) | Systematic or narrative review (protein source and quality of RUTF) |
| Manary 2016 (14) | Systematic or narrative review (protein source and quality of RUTF) |
| Ndekha 2005 (59) | No intervention of RUTF containing less than 50% of protein from dairy products (standard RUTF in home setting) |
| Nga 2013 (60) | No intervention of RUTF containing less than 50% of protein from dairy products (RUTF with similar milk composition) |
| Owino 2014 (61) | Infants/children without SAM (healthy school-aged children) |
| Sandige 2004 (62) | No intervention of RUTF containing less than 50% of protein from dairy products (RUTF with similar milk composition) |
| Schlossman 2018 (63) | Adults or older children(school age children and adults) and infants/children without SAM |
| Shewade 2013 (64) | No intervention of RUTF containing less than 50% of protein from dairy products (RUTF with similar milk composition) |
| Sigh 2018 (65) | No child outcomes evaluated (acceptability study) |
| Thapa 2017 (66) | No intervention of RUTF containing less than 50% of protein from dairy products (RUTF with similar milk composition) |
| Weber 2017 (67) | Infants/children without SAM (children with MAM included) |

**Supplemental Figure 1.** Subgroup meta-analysis comparing non-dairy and low dairy versions of ready-to-use therapeutic food in terms of the rate of weight gain in grams per kilogram of body weight per day.


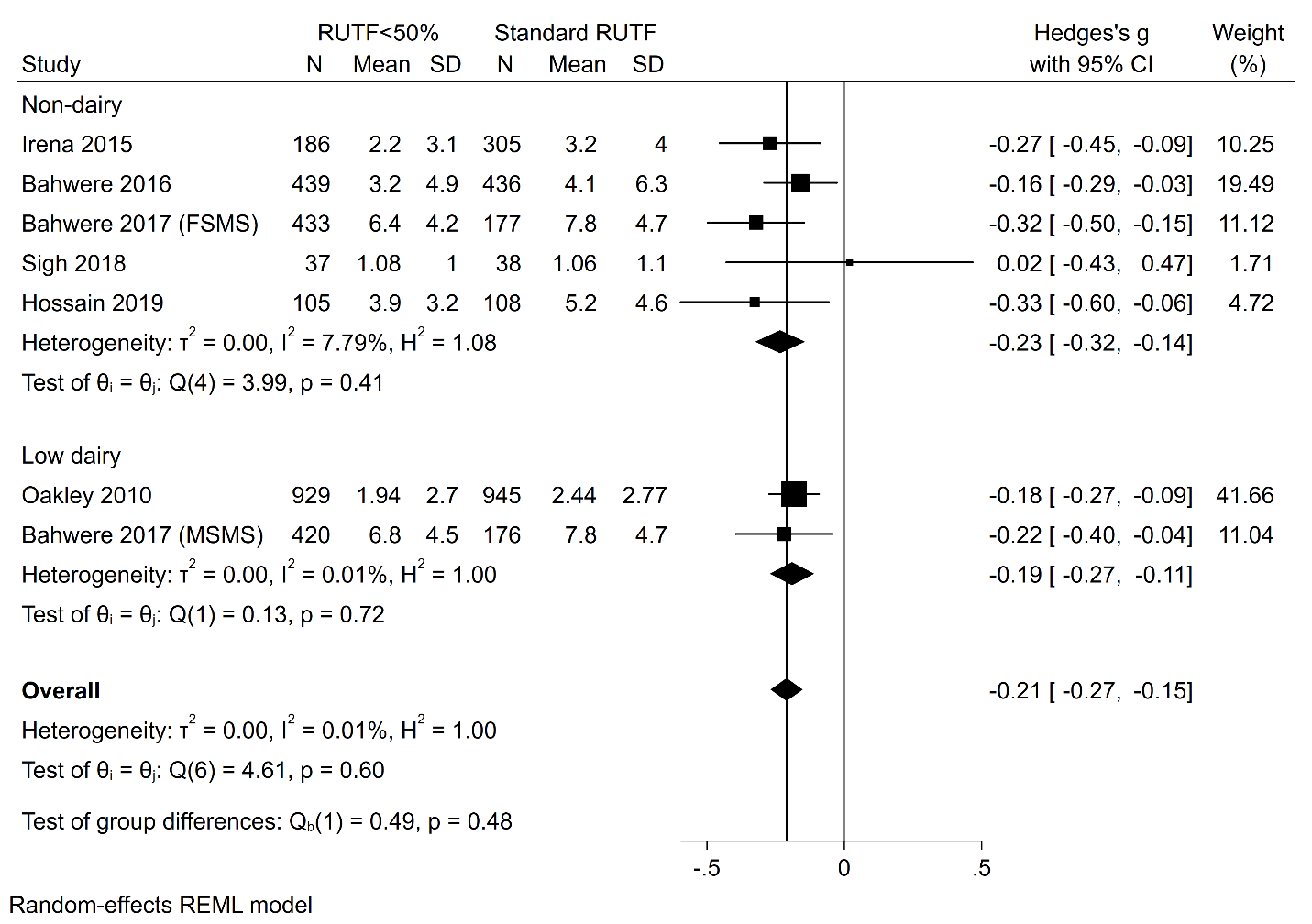


RUTF<50% represents RUTF with less than 50% of protein coming from dairy products; REML: restricted maximum likelihood; RUTF: ready-to-use therapeutic food.

**Supplemental Figure 2.** Network meta-analysis comparing non-dairy and low dairy versions of ready-to-use therapeutic food in terms of the rate of weight gain in grams per kilogram of body weight per day.


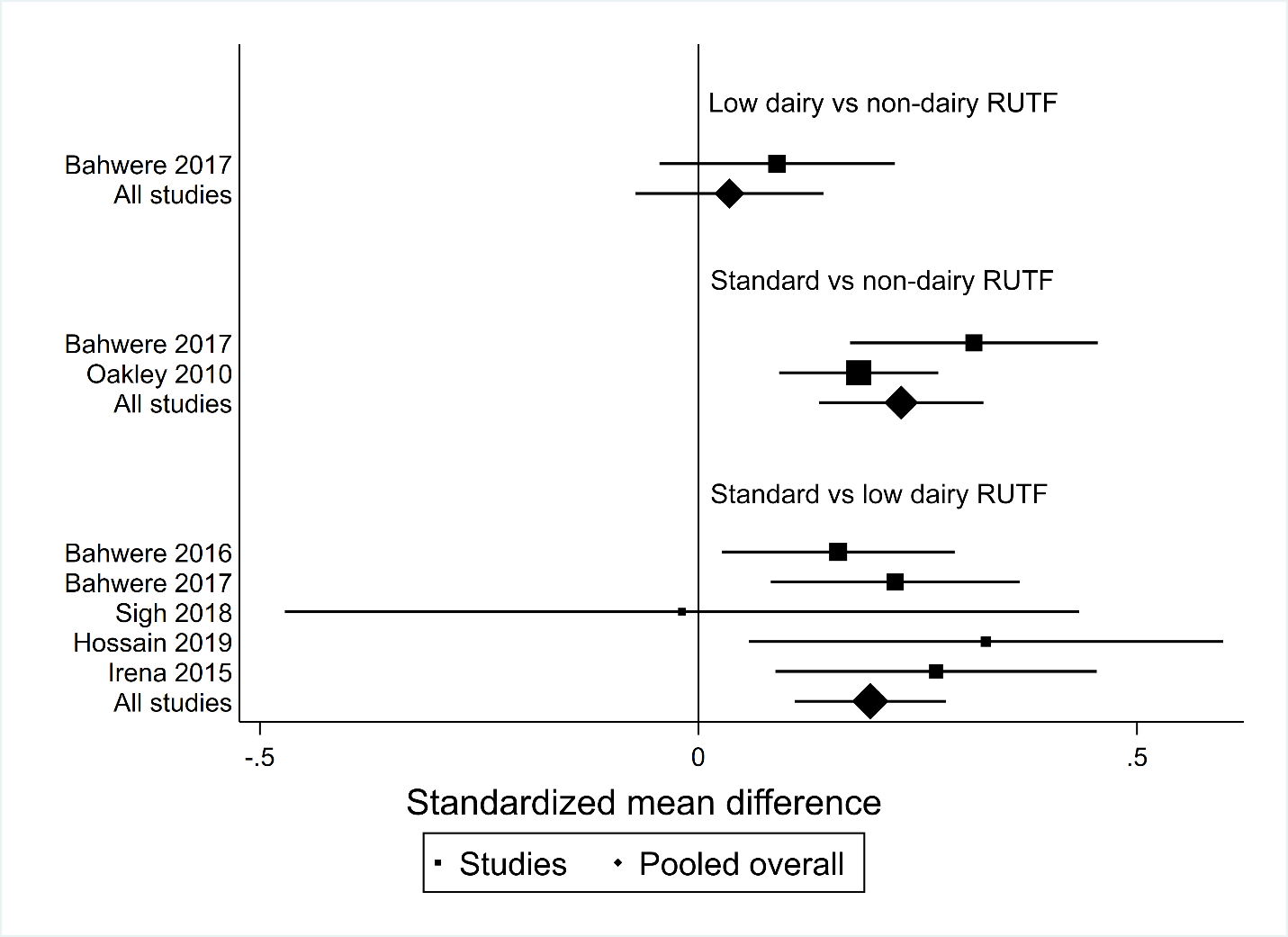


RUTF: ready-to-use therapeutic food.

**Supplementary Figure 3.** Meta-analysis of time to recovery in days.

**
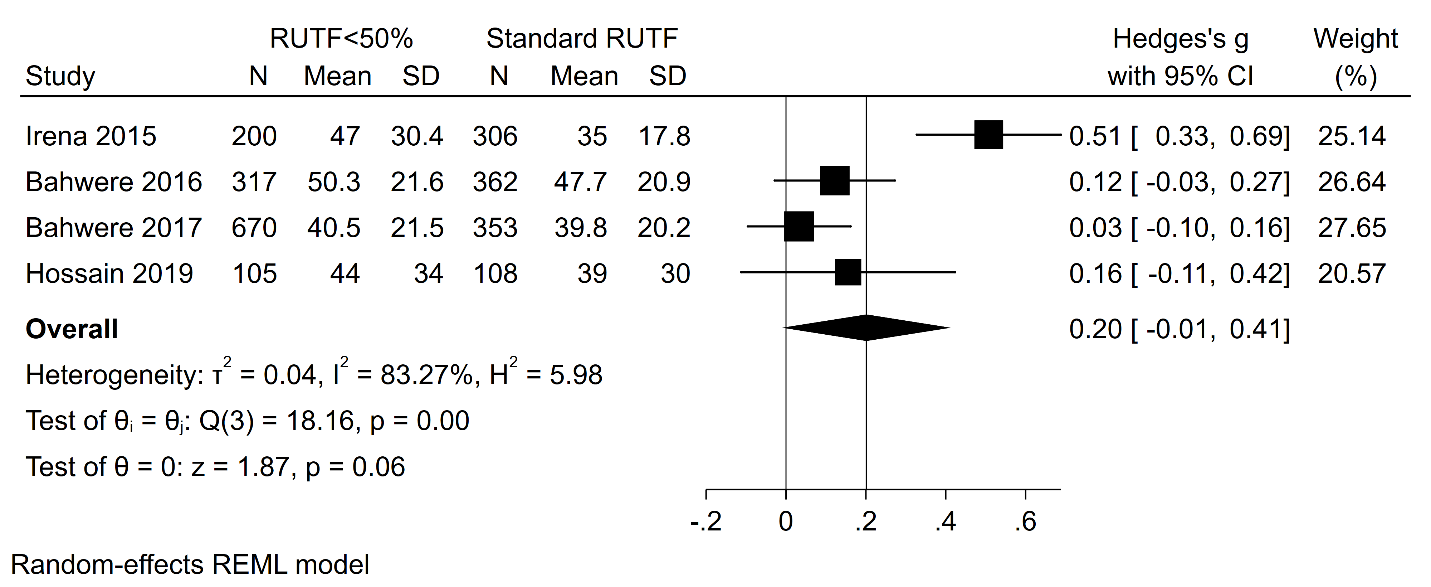
**

RUTF<50% represents RUTF with less than 50% of protein coming from dairy products; REML: restricted maximum likelihood; RUTF: ready-to-use therapeutic food.

**Supplementary Figure 4.** Meta-analysis of weight-for-height z-scores.

**
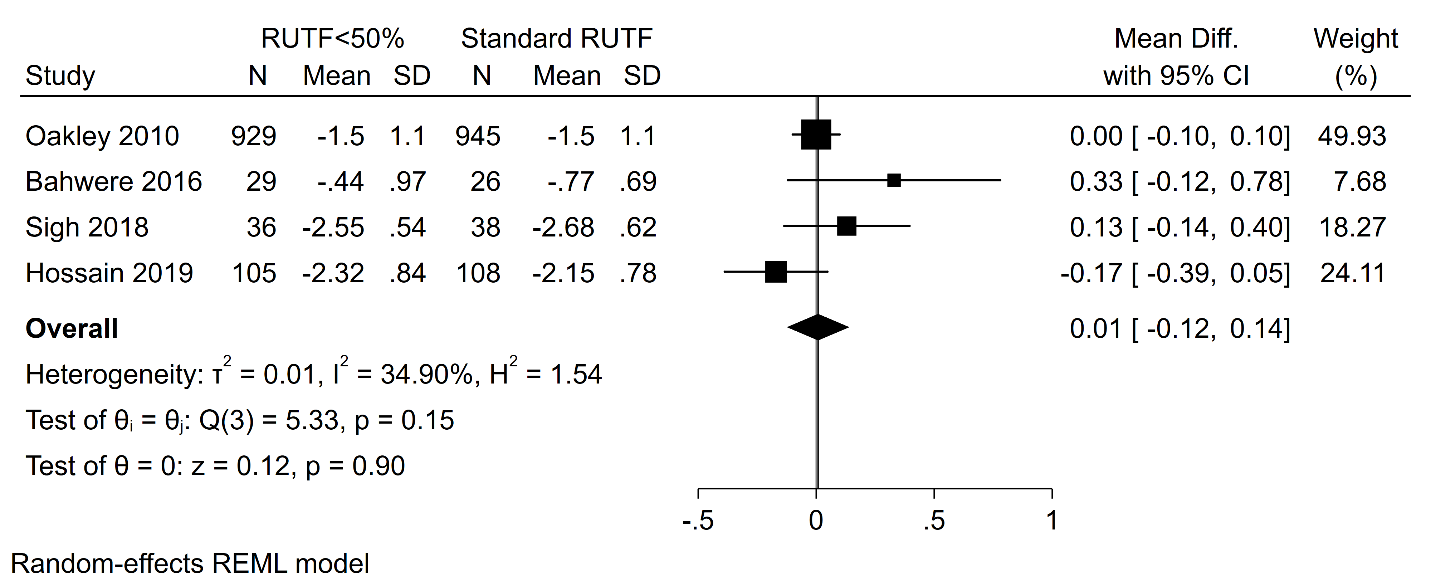
**

RUTF<50% represents RUTF with less than 50% of protein coming from dairy products; REML: restricted maximum likelihood; RUTF: ready-to-use therapeutic food.

**Supplementary Figure 5.** Meta-analysis of mid-upper arm circumference.

**
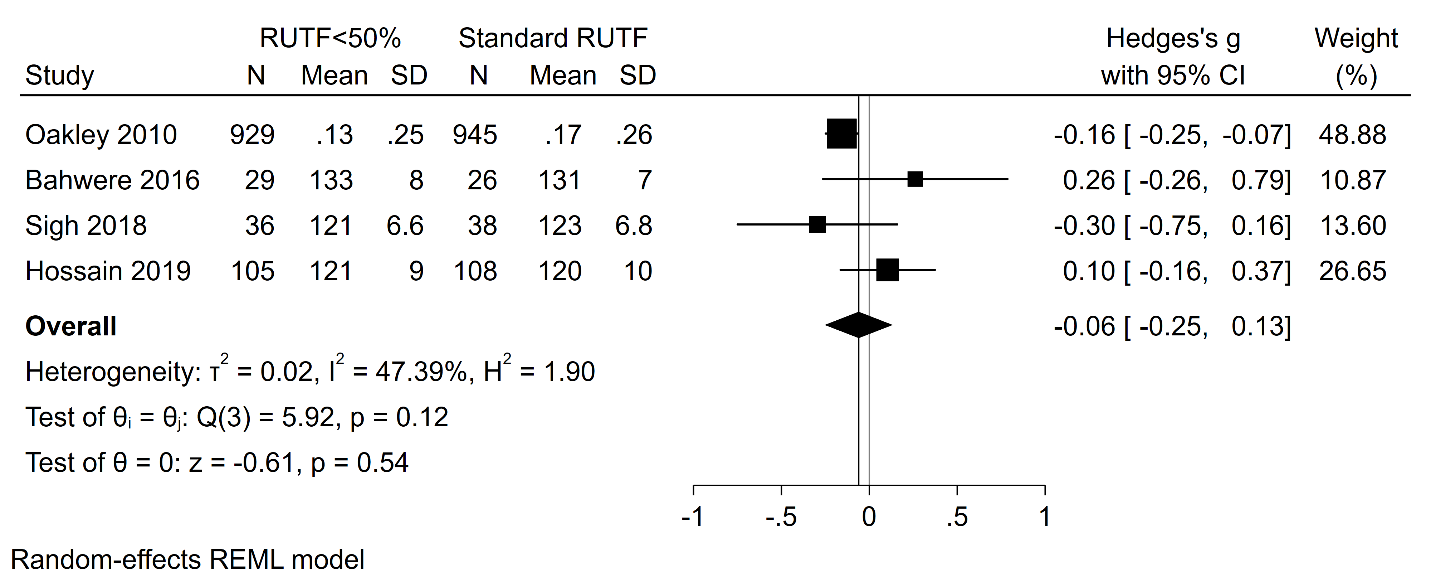
**

RUTF<50% represents RUTF with less than 50% of protein coming from dairy products; REML: restricted maximum likelihood; RUTF: ready-to-use therapeutic food.

**Supplementary Figure 6.** Meta-analysis of weight-for-age z-scores.

**
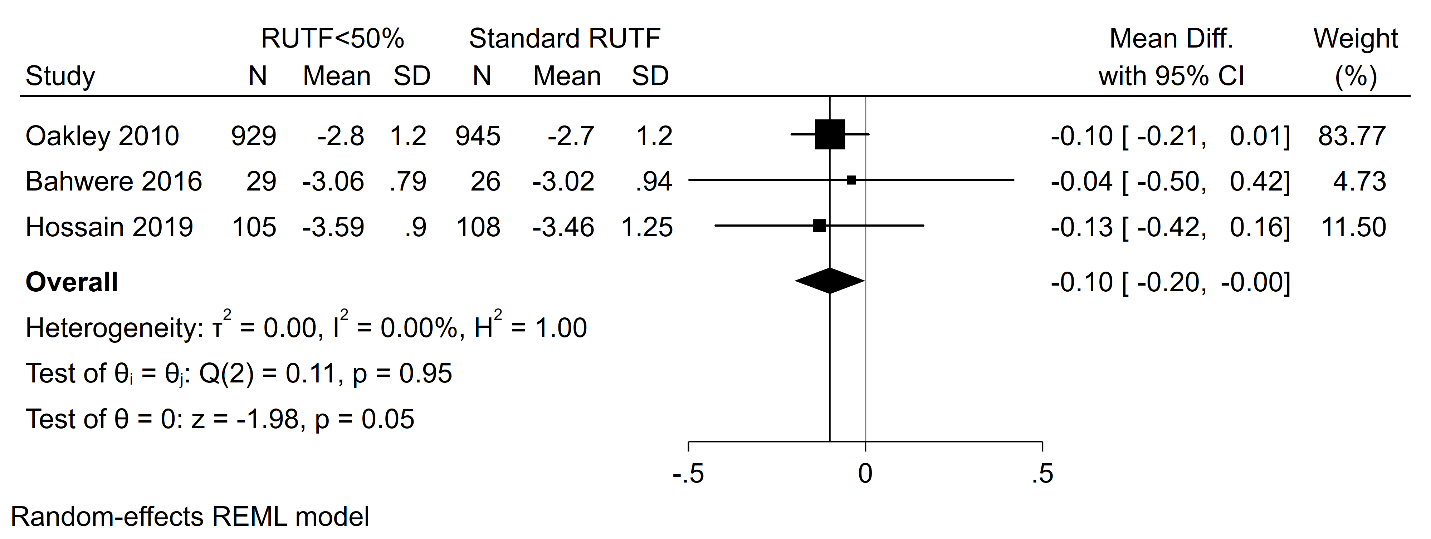
**

RUTF<50% represents RUTF with less than 50% of protein coming from dairy products; REML: restricted maximum likelihood; RUTF: ready-to-use therapeutic food.

**Supplementary Figure 7.** Meta-analysis of height-for-age z-scores.

**
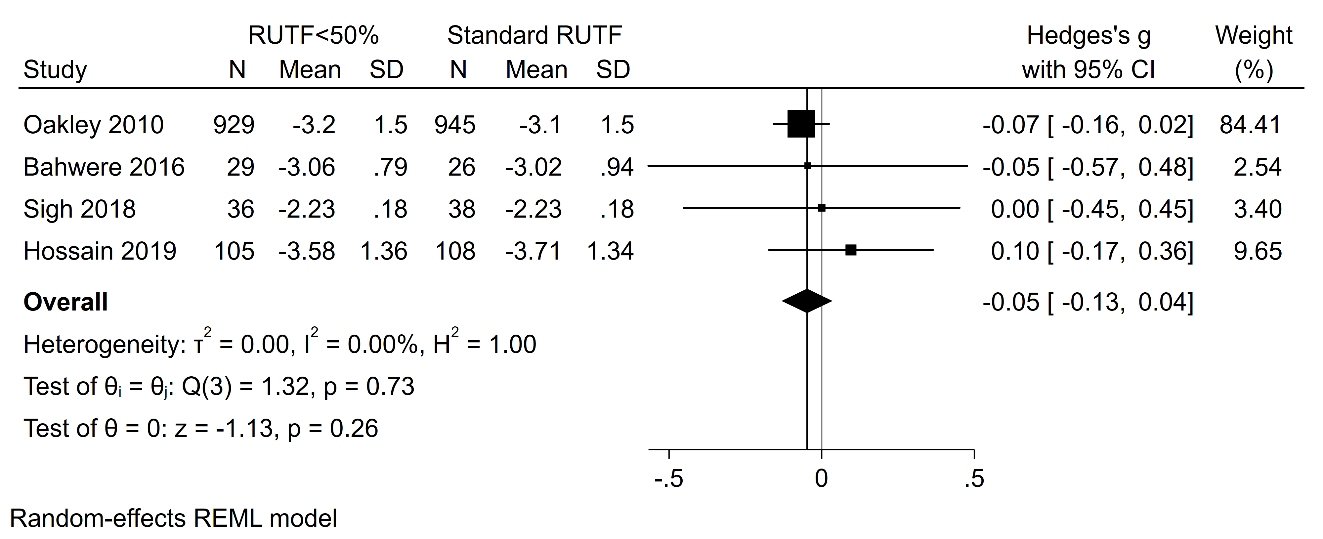
**

RUTF<50% represents RUTF with less than 50% of protein coming from dairy products; REML: restricted maximum likelihood; RUTF: ready-to-use therapeutic food.
